# Effect of Fertilization on Phenolics of Rapeseeds and Their Antioxidant Potential

**DOI:** 10.3390/foods13040561

**Published:** 2024-02-12

**Authors:** Ryszard Amarowicz, Bożena Cwalina-Ambroziak, Michał Adam Janiak, Marta Damszel, Arkadiusz Stępień, Katarzyna Sulewska, Magdalena Karamać, Kamila Penkacik

**Affiliations:** 1 Institute of Animal Reproduction and Food Research, Polish Academy of Sciences, 10 Tuwima Street, 10-748 Olsztyn, Poland; m.janiak@pan.olsztyn.pl (M.A.J.); k.sulewska@pan.olsztyn.pl (K.S.); m.karamac@pan.olsztyn.pl (M.K.); k.penkacik@pan.olsztyn.pl (K.P.); 2 Department of Entomology, Phytopathology and Molecular Diagnostics, Faculty of Agriculture and Forestry, University of Warmia and Masury, 11-041 Olsztyn, Poland; bambr@uwm.edu.pl (B.C.-A.); marta.damszel@uwm.edu.pl (M.D.); 3 Department of Agroecosystems and Horticulture, Faculty of Agriculture and Forestry, University of Warmia and Mazury, 10-721 Olsztyn, Poland; arkadiusz.stepien@uwm.edu.pl

**Keywords:** rapeseed, fertilization, phenolic compounds, antioxidant activity, HPLC

## Abstract

Three varieties of rapeseed (Castilla, California, and Nelson F1) were cultivated using medium–intensive (control), intensive, and economical (spare) technologies with different nitrogen and sulfur fertilization techniques. The antioxidant potential of rapeseeds was investigated using ABTS, FRAP, and DPPH assays. The content of total phenolic compounds was determined using the Folin–Ciocalteu phenol reagent. The profile of phenolic compounds was determined using high-performance liquid chromatography (HPLC). Diversifying fertilization in various ways influenced the content of phenolic compounds in extracts of rapeseed. In extracts from the Nelson F1 rapeseeds, intensive cultivation resulted in a lower content of phenolic compounds compared to the control group. Economic fertilization reduced the content of phenolic compounds in seeds from the California variety. HPLC chromatograms of the extracts were characterized by the presence of five (California and Castilla) and six (Nelson F1) main phenolic compounds. Two compounds were identified as sinapine and sinapic acid; others were classified as derivatives of sinapic acid. The effect of fertilization on the antioxidant activity of the seeds and their extracts varied depending on the plant variety and antioxidant assay. For the Castilla and California varieties, no differences were found in the results of the ABTS assay. The antiradical activity against ABTS^•+^ of extracts from the Nelson F1 intensive and spare cultivated seeds was higher than that of extracts from control seeds. The FRAP values of extracts/seeds from the Castilla variety cultivated using different methods did not differ significantly. The results of the DPPH assay were not affected by fertilization in the case of extracts from the California and Castilla varieties. However, the extracts from spare cultivated seeds of Nelson F1 exhibited stronger antiradical activity against DPPH^•^. These findings highlight the complex relationship between fertilization practices, phenolic compound accumulation, and antioxidant activity in rapeseed. Integrating varietal traits and cultivation practices is crucial for optimizing the nutritional benefits of rapeseed.

## 1. Introduction

Phenolic compounds are widely distributed in the plant world. They differ in their chemical structure, which in turn affects their chemical properties and biological activity. They appear in all parts of plants, usually accumulated in cell vacuoles, paratomatal, epidermal, subepidermal leaf cells, shoots, and hairs [1], and take part in many plant life activities such as morphogenesis, sex determination, photosynthesis, respiration, regulation of gene expression, and regulation of the synthesis of growth hormones. One of the defense mechanisms of plants is the production of phenolics, which protect against herbivore attacks but also against pathogens and many abiotic stress factors. Many insects are sensitive to the effects of phenolic acids, flavonoids, tannins, and lignans [2,3]. Phenolics exist in various forms, such as soluble-free, soluble-bound, including esterified, etherified, or glycosylated, and insoluble-bound forms, based on their association with food matrices in plants [4].

The diverse biological properties of phenolics have resulted in a sharp increase in interest in them as bioactive food ingredients, which, as nutraceuticals, can contribute to the therapy of various disorders such as neurological diseases and cancer. Most phenolic compounds exhibit strong antioxidant properties. The antioxidant activity of phenolic compounds depends on their chemical structure and molecular weight and may occur through various mechanisms of action, including electron or hydrogen atom transfer, chelation of the pro-oxidant metal ions, and inhibition of enzymes that catalyze oxidation reactions [5].

Phenolic compounds with the ability to scavenge free radicals may participate in the non-enzymatic antioxidative protection system of the human body. Free radicals can be formed between others under the influence of xenobiotics, UV radiation, and ionizing radiation. Mutual interaction of free radicals with cellular biomolecules such as nucleic acids, proteins, lipids, and carbohydrates leads to various structural damages, for example in the DNA strand, which in turn results in mutations. The resulting changes in DNA may be a signal to initiate pathological cell proliferation and the cancer process. Free oxygen species and free radicals can also contribute to the development of many civilization diseases, for example, atherosclerosis, Parkinson’s and Alzheimer’s diseases, obesity, and chronic inflammation [6].

Rapeseed (*Brassica napus* L.) seeds are a rich source of oil, protein, and phenolic compounds [7,8,9,10]. Sinapine, the choline ester of sinapic acid, is the dominant phenolic compound of rapeseed. Sinapic acid in rapeseed also occurs as free phenolic acids or glycosides [11]. In rapeseed, small amounts of other phenolic acids, such as ferulic acid, caffeic acid, *p*-coumaric acid, vanillic acid, syringic acid, *p*-hydroxybenzoic acid, gentisic acid, and protocatechuic acid, have been reported [9]. The major phenolic compound of rapeseed oil is canolol [12], which is produced by the decarboxylation of sinapic acid during rapeseed thermal processing [13]. The presence of condensed tannins in rapeseed and canola hulls was reported by Naczk et al. [14] and Amarowicz et al. [15].

The antioxidant activity of phenolic compounds from rapeseed or canola was reported by several authors and determined through different methods showing free radical scavenging activity and reducing abilities of these compounds [15,16,17,18,19,20]. Extracts of phenolic compounds obtained from rapeseed were stronger than those of legumes and cereals [5].

The important accumulation of phenolic compounds in rapeseed results from climatic and soil conditions [21]. Utilizing appropriate production technologies to cultivate plants is an important element in modern, sustainable agriculture, and there are several goals to it, including optimizing mineral fertilization and protecting plants against pests. Nitrogen [22] and sulfur [23] fertilization is very important for many species, not only Brassicaceae plants; however, excess fertilization is harmful and impacts the cost of growing crops [24]. Together with the smaller yields of *Brassica rapa* L. chinensis, the ecological system showed a significantly higher level of phenolic compounds in plants compared to plants cultivated using the conventional system. According to Zhao et al. [25], this may result from the unavailability of nutrients.

The effect of fertilization on the plant phenolic compounds is not easily predictable, and the explanation of the different behaviors is not univocal or understandable on the basis of a biochemical pathway. The experiments of the effect of fertilization on the content of phenolic compounds in fruits (apple, black current, blueberry, grapes, marionberry, orange, peach, strawberry, yellow plum) and vegetables (cabbage, carrot, chicory, eggplant, lettuce, onion, pepper, potato, red beat, spinach, tomato) were finished with conclusions of “No differences between organic and conventional production”, “Higher in biodynamic than in conventional production”, and “Higher in organic than in conventional production”. The carbon/nitrogen balance (CNB) pathways can be taken into account when studying the effect of nitrogen fertilization on the content and profile of plant phenolic compounds [26,27,28].

This research aimed to determine the effect of agro-technology (different fertilization) on the antioxidant potential of rapeseeds and their phenolic profile. There are no publications on this important topic in the Scopus and Web of Science databases.

The aim of this research was related to the role of rapeseed as an oil plant and the possibility of using the material after oil extraction as a product to obtain natural oxidants.

## 2. Materials and Methods

### 2.1. Plant Material

Winter rape was cultivated in a 4-year monoculture in a close-field experiment in a random block system in triplicate. The experiment was performed in the Agricultural Production Plant in Bałcyny near Olsztyn in Poland (53°78′ N, 20°48′ E) on soil of a granulometric composition of loamy sand with a high content of phosphorus and an average amount of potassium and magnesium.

The following varieties of winter oilseed rape were included: two population varieties, California (tolerant of fungal diseases, with very good winter hardiness, moderately early in ripening) and Castilla (tolerant of fungal diseases, with very good winter hardiness, with even maturation of siliques), and the Nelson F1 hybrid variety (moderately resistant to fungal diseases, good winter hardiness, medium-early in ripening). Based on previous research [29,30], the following cultivation technologies were applied: medium–intensive (control), intensive, and economical (spare), differing in nitrogen and sulfur fertilization. The agrotechnical conditions of the experiment are shown in Table 1. Seeds were collected at the technical maturity stage.

### 2.2. Chemicals

All solvents used were of analytical grade. Methanol, *n*-hexane, ferrous chloride, sinapic acid, sinapine, the Folin–Ciocalteu phenol reagent, 2,2-diphenyl-1-picrylhydrazyl (DPPH) radical, 2,2′-azinobis-(3-ethylbenzothiazoline-6-sulphonic acid) (ABTS), 2,4,6-tri(2-pyridyl)-*s*-triazine (TPTZ), and 6-hydroxy-2,5,7,8-tetramethyl-chroman -2-carboxylic acid (Trolox) were obtained from Sigma (St. Louis, MO, USA).

### 2.3. Extraction Procedure

Seeds were ground and defatted using a Soxhlet extractor and n-hexane as a solvent. Phenolic compounds present in defatted plant material were extracted with a methanol and water mixture (8:2; *v*/*v*) for 30 min at a temperature of 50 °C. The ratio of plant material to solvent was 1:10 (*w*/*v*) [31]. Extraction was carried out in a shaking water bath (Elpan 357, Wrocław, Poland). The extracts were filtrated, and the solid residue was extracted twice more. For the evaporation of methanol from the combined filtrates, a Büchi R-200 rotary evaporator (Büchi, Flawil, Switzerland) was used. The water residue was removed using a freeze dryer.

### 2.4. Determination of Total Phenolics

The total phenolic content in the rapeseed extracts was estimated using a colorimetric method with the Folin–Ciocalteu phenol reagent [32]. Briefly, a 0.25 mL aliquot of extract dissolved in methanol was added to a test tube containing 4 mL of distilled water. Then, 0.25 mL of the Folin–Ciocalteu phenol reagent and 0.25 mL of a saturated Na_2_CO_3_ solution were added. After 30 min, the absorbance was recorded at 725 nm using a Beckman DU 7500 diode array spectrophotometer (Beckman Coulter, Inc., Brea, CA, USA), and sinapic acid was used to prepare the standard curve. The results were expressed as mg of sinapic acid equivalents (SAE) per g of extract or g of seed fresh matter (FM).

### 2.5. Trolox Equivalent Antioxidant Capacity Determination

The ABTS assay described by Re et al. [33] was used to measure the Trolox equivalent antioxidant capacity (TEAC). The solution of ABTS^•+^ was prepared by mixing an ABTS solution in water with 2.45 mM sodium persulfate. After shaking for 12 h at room temperature, the obtained ABTS^•+^ stock solution was diluted with methanol to the desired concentration, giving an absorbance of 0.720 at 734 nm. For the colorimetric determination, the absorbance of the sample containing 2 mL of the ABTS^•+^ solution and 20 µL of rapeseed extract was read after 10 min at 734 nm. The calibration curve was plotted using the Trolox standard. The results were expressed as mmol of Trolox equivalents (TE) per g of extract or g of seed FM.

### 2.6. Ferric-Reducing Antioxidant Power Determination

A ferric-reducing antioxidant power (FRAP) assay was carried out according to the procedures of Benzie and Strain [34] and Amobonye et al. [35]. For the preparation of the working FRAP reagent, 300 mM acetate buffer (pH 3.6) was mixed with 10 mM TPTZ (in 40 mM HCl) and 20 mM FeCl_3_ × 6 H_2_O in a ratio of 10:1:1 (*v*/*v*/*v*). For the colorimetric determination, the absorbance of the sample containing 2.25 mL of the FRAP reagent, 75 μL of methanolic solution of rapeseed extract, and 225 μL of deionized water was read at 593 nm after 30 min of incubation. The FRAP values were calculated using the calibration curve for FeSO_4_. The results were expressed as mmol Fe^2+^ equivalents per g of extract or g of seed FM.

### 2.7. Determination of DPPH Radical Scavenging Activity

The antiradical activity of the extracts against DPPH^•^ was determined according to the Brand-Williams et al. [36] method, with modifications proposed by Sulewska et al. [37]. A 2 mL of deionized water was mixed with a 0.1 mL methanolic solution containing between 0.47 and 2.35 mg of rapeseed extract. Then, 0.25 m of 1 mM DPPH^•^ solution and 2 mL of deionized water were added. After 20 min, the absorbance of the solution was read at 517 nm. The curves of absorbance vs. extract concentration in the reaction mixture were plotted. The results were expressed as the half maximal scavenging concentration (SC_50_)—the concentration of antioxidants that scavenges half of the radicals.

### 2.8. HPLC Analysis

Rapeseed extract methanolic solution (5 mg/mL) was filtered (0.45 μm cellulose acetate filter; Millipore, Burlington, MA, USA), and phenolic compounds were analyzed using a Shimadzu HPLC system (Shimadzu Corp., Kyoto, Japan). This system consisted of two LC-10AD pumps, an SPD-M 10A photodiode array detector, and an SCL 10A system controller. Phenolic compounds were separated on a prepacked Luna C_18_ column (4 × 250 mm, 5 μm; Phenomenex, Torrance, CA, USA) for 50 min in a gradient system of 5–40% (*v*/*v*) acetonitrile in water adjusted to pH 2.5 with trifluoroacetic acid, at a flow rate of 1 mL/min [38]. The separation was monitored at 320 nm, and the injection volume was 20 μL. The content of sinapine and sinapic acid was calculated using the external standard method. The content of other phenolic compounds showing UV-DAD spectra typical for sinapic acid was expressed as equivalents of this acid.

### 2.9. Statistical Analysis

In this study, three samples of each rapeseed variety from each field variant were analyzed. Moreover, chemical determinations were triplicated. Results were reported as the mean and standard deviation. For evaluation of the significance of differences among means, analyses of variance (ANOVA) and the Duncan’s post hoc test were performed at a level of *p* < 0.05 (GraphPad Prism version 6.04; GraphPad Software, San Diego, CA, USA). The principal component analysis (PCA) and Pearson correlation analysis were carried out using the Statistica 13.1 software (StatSoft Corp., Kraków, Poland).

## 3. Results

The content of total phenolic compounds in the rapeseed extracts ranged from 48.3 mg SAE/g extract (Castilla, intensive) to 60.9 mg SAE/g extract (California, control) (Table 2). The results expressed in relation to seeds ranged from 4.47 mg SAE/g seed FM (Castillo, spare) to 6.04 mg/g seed FM (Nelson F1, control). The results obtained for the three rapeseed varieties (control) varied as follows: California > Nelson F1 > Castilla for extracts, and Nelson F1 > California > Castilla for seed FM. Diversified fertilization in various ways influenced the total phenolic content of extracts/seeds from different cultivars. For extracts from the Nelson F1 rapeseeds, intensive fertilization resulted in a lower total phenolic content compared to the control group. Economic fertilization reduced the content of total phenolic compounds in extracts of the California variety. The total phenolic content in the California seeds was higher for the intensive group than for the spare group (Table 2).

The antioxidant activity determined using ABTS and FRAP assays also depended on the rapeseed variety (Table 2). The Castilla samples showed lower TEAC compared to the seeds and extracts of the other varieties. FRAP values were higher for the California extracts. When considering the effect of fertilization on TEAC, no differences were found between extracts for individual varieties and between seeds of different groups for the California and Castilla varieties. In the case of the Nelson F1 variety, the TEAC of seeds from the intensive and spare groups (0.043 mmol TE/g seed FM) was higher than that of seeds from the control group (0.033 mmol TE/g seed FM). The obtained FRAP values for extracts of the Castilla variety did not differ from each other. In the California variant, the FRAP values for the spare group (1.43 Fe^2+^/g extract; 0.115 mmol Fe^2+^/g seed FM) were lower than the FRAP values for the control group (1.61 mmol Fe^2+^/g extract; 0.138 mmol Fe^2+^/g seed FM) and the intensive group (1.66 mmol Fe^2+^/g extract; 0.144 mmol Fe^2+^/g seed FM).

**Figure 1:**
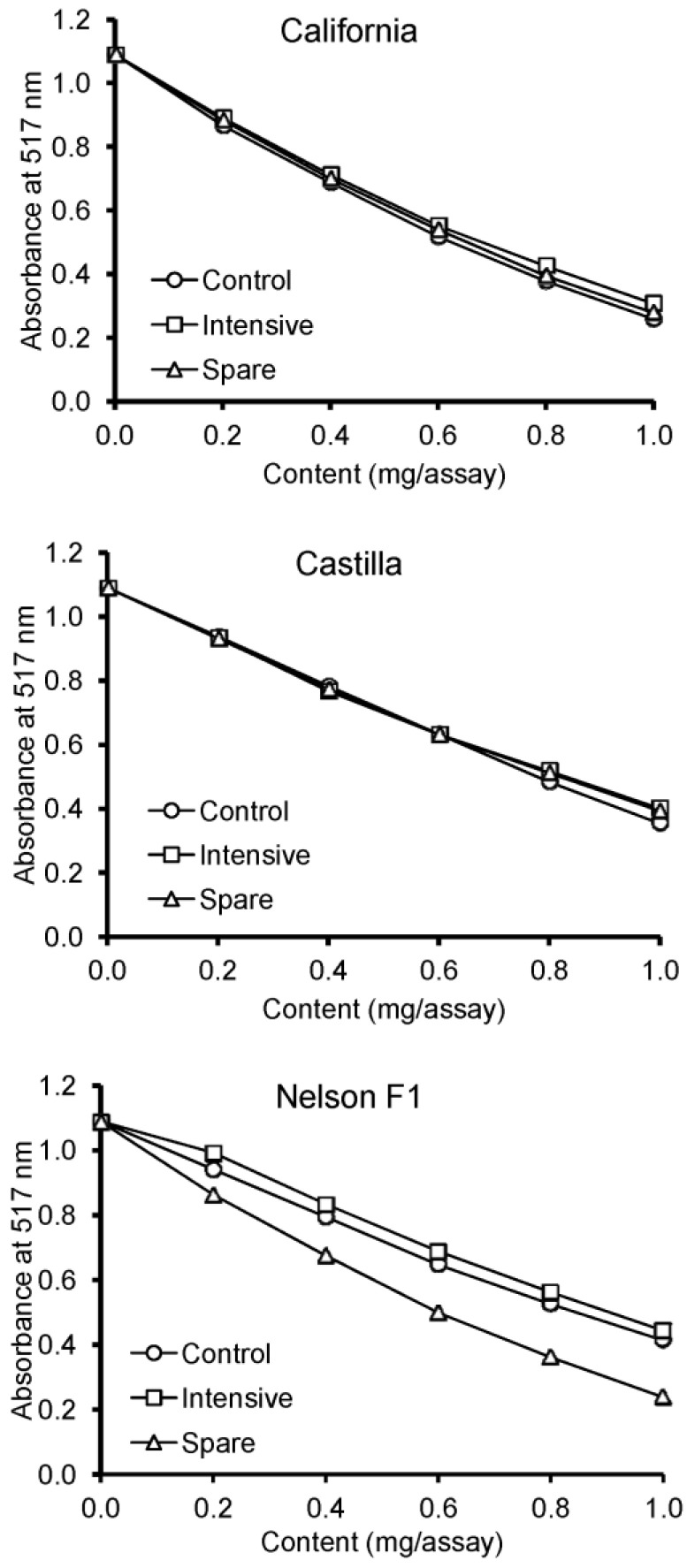
Antiradical activity of rapeseed extracts against DPPH radicals.

**Figure 2:**
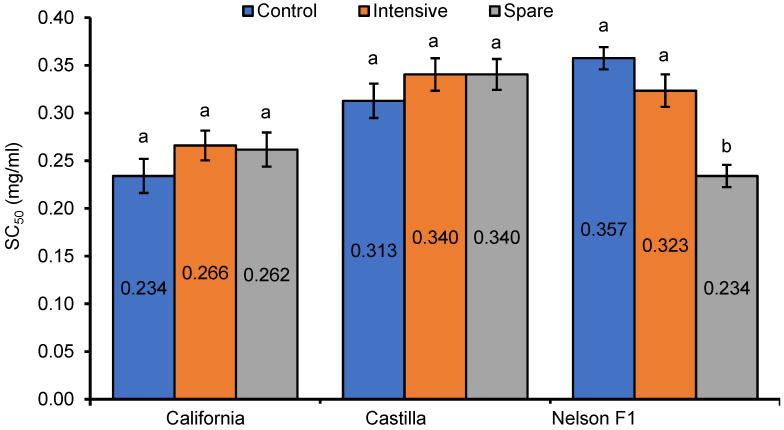
Antiradical activity of rapeseed extracts against DPPH radicals expressed as SC_50_. Means with different letters are significantly different (*p* < 0.05).

The results of the DPPH assay are depicted in Figure 1 and Figure 2. For all extracts, dose-related antiradical activity against DPPH^•^ was observed. Comparing SC_50_ values, the effect of fertilization was not observed for extracts of the California and Castilla varieties. In turn, the extracts of the seeds from the Nelson F1 spare group exhibited stronger antiradical activity against DPPH^•^ than the extracts from the intensive and control groups.

**Figure 3:**
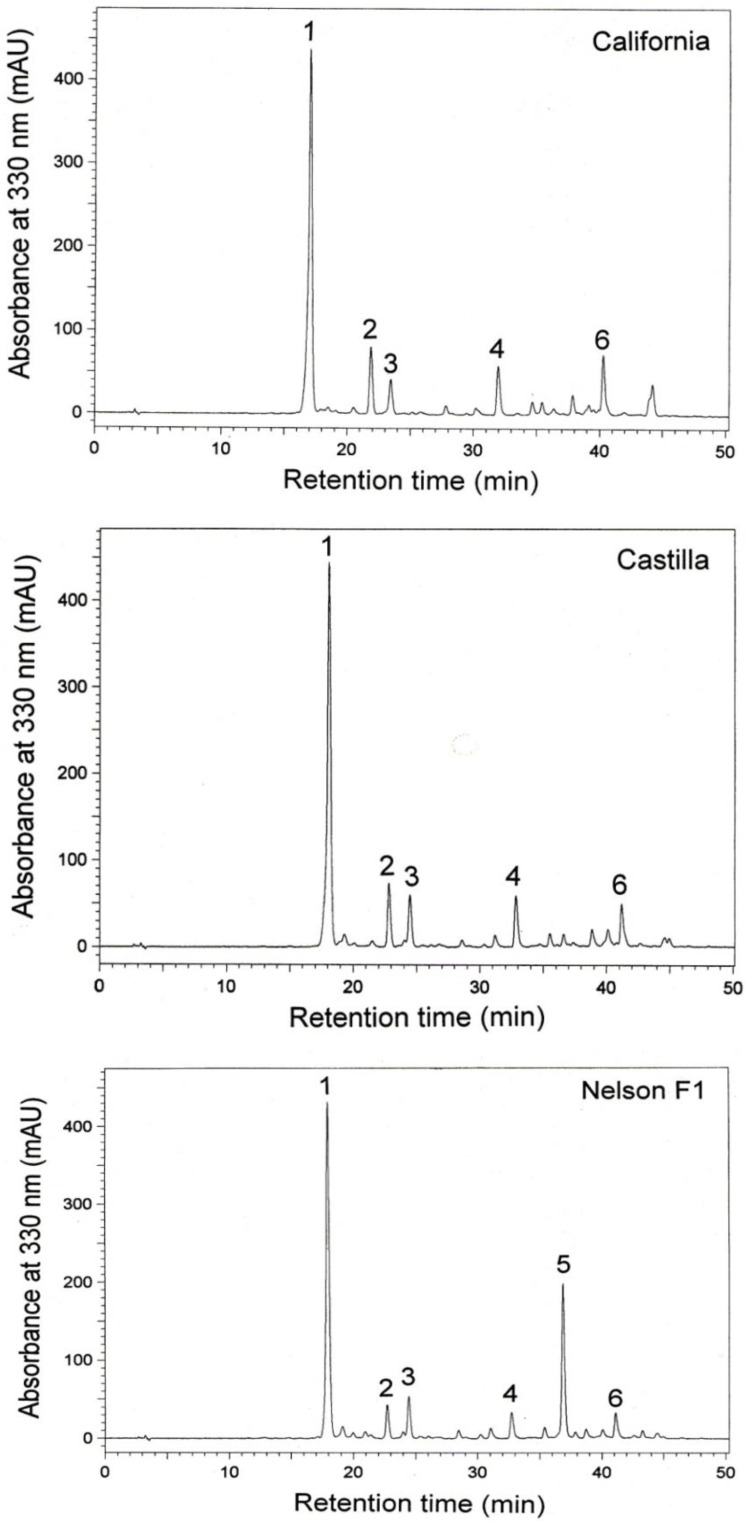
HPLC chromatograms of rapeseed extracts. The peak numbers correspond to the compounds listed in Table 3.

The HPLC chromatograms of the extracts were characterized by the presence of five (California and Castilla) and six (Nelson F1) main phenolic compounds (Figure 3). Applying the original standards, compounds 1 and 4 were identified as sinapine and sinapic acid, respectively. Based on the DAD-UV spectra, it was tentatively found that compounds 2, 3, 5, and 6 were derivatives of sinapic acid.

The content of sinapine in the rapeseed extracts ranged from 64.5 mg/g extract (Nelson F1 intensive group) to 81.9 mg/g extract (California intensive group) (Table 3). The lowest content of sinapic acid was determined in the extracts from the Nelson F1 control group—3.8 mg/g extract, and the highest was in the extracts from the California control group—6.3 mg/g extract. The content of sinapine in seeds ranged from 6.2 mg/g seed FM (California spare group) to 7.4 mg/g seed FM (Nelson F1, control group). The lowest content of sinapic acid was found in the Nelson F1 intensively cultivated seeds—0.41 mg/g seed FM. The control and intensively cultivated seeds of the California and Castillo seeds in the control group were characterized by the highest content of sinapic acid—0.54 mg/g seed FM. Interestingly, compound 5 was quantified only in the seeds/extracts of the Nelson F1 cultivar. The content of this compound was relatively high in the range of 16.9–18.3 mg/g extract and 1.69–2.05 mg/g seed FM. Unlike sinapine and sinapic acid, for which no effect of fertilization was found, its content in control seeds was significantly higher than in intensively cultivated and spare seeds.

**Figure 4:**
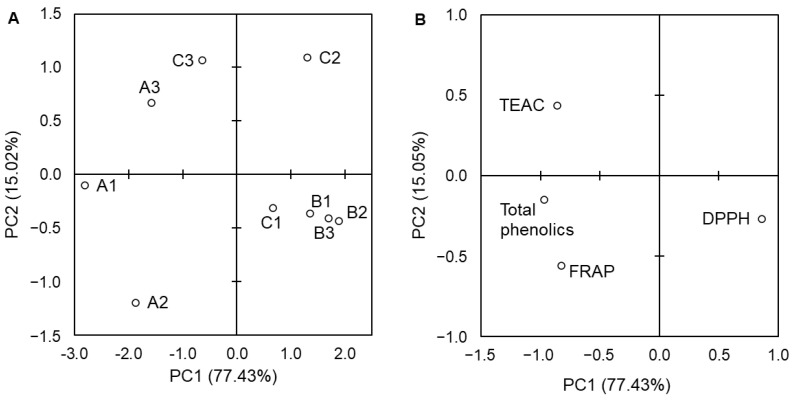
Plots of principal component analysis (PCA) of the objects (**A**) and variables (**B**) for data relating to extracts. A1—California control group; A2—California intensive group; A3—California spare group; B1—Castilla control group; B2—Castilla intensive group; B3—Castilla spare group; C1—Nelson F1 control group; C2—Nelson F1 intensive group; C3—Nelson F1 spare group; TEAC—Trolox equivalent antioxidant capacity; FRAP—ferric-reducing antioxidant potential; DPPH—DPPH radical scavenging activity.

**Figure 5:**
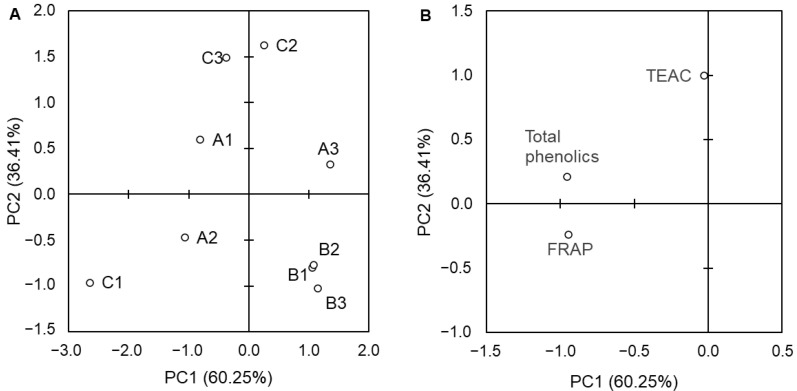
Plots of principal component analysis (PCA) of the objects (**A**) and variables (**B**) for data relating to seed FM. A1—California control group; A2—California intensive group; A3—California spare group; B1—Castilla control group; B2—Castilla intensive group; B3—Castilla spare group; C1—Nelson F1 control group; C2—Nelson F1 intensive group; C3—Nelson F1 spare group; TEAC—Trolox equivalent antioxidant capacity; FRAP—ferric-reducing antioxidant potential.

The collected data from the analyses of the total phenolic content and antioxidant assays were subjected to PCA to identify the relationships between these variables and rapeseed varieties cultivated in different ways. The PCA results computed for the values of variables expressed per extract are depicted in Figure 4 and for those expressed per seed FM in Figure 5. In the case of data relating to extracts, the first two components (PC1 and PC2) explained 92.45% of the total variance. The system was divided along PC1 into two groups. The first was formed by extracts from the California variety seeds cultivated in each method (A1–A3) and extracts from the Nelson F1 spare cultivated seeds (C3). Most of the variables (total phenolic content and results of ABTS and FRAP assays) described this group. The second group was formed by the rest of the samples, which were closely associated with the results of the DPPH assay. For data relating to seed FM, PC1 and PC2 significantly explained 96.66% of the total variance. Variables affected the A1 and A2, as well as seeds of the Nelson F1 variety from the control (C1) and spare (C3) cultivations. To determine direct correlations between variables, a Pearson correlation analysis was performed for data relating to the extracts. Total phenolic content significantly (*p* < 0.05) correlated with the results of the ABTS and FRAP assays (Table 4). A high correlation coefficient (0.672; *p* < 0.05) was also found for the correlation between TEAC and FRAP. These findings are in line with those shown by PCA (Figure 4B).

## 4. Discussion

The results of total phenolic and sinapine content in rapeseed extracts and seeds are comparable with those reported in the literature. Thiyam et al. [20] reported that the content of total phenolics ranged from 12 to 24 mg/g of rapeseed oil-free meal. According to Mert-Türk et al. [39], the sinapine content in five rapeseed cultivars ranged from 0.33 to 0.43 g/kg. Similar results were reported for sinapic acid content in rapeseed by Siger et al. [40], who found a significant variation in the content of this phenolic compound in rapeseed between the individual harvest years within the same varieties. The variety was a very important factor that influenced the content of phenolic compounds in broccoli [41,42].

Sinapine and sinapic acid are the well-known phenolic compounds of rapeseed. In our research, we also determined four other rapeseed phenolic compounds that were tentatively identified as sinapic acid derivatives. Identification was made based on the similarity of the shape of the UV-DAD spectra with a maxima at a wavelength of 328–330 nm of these compounds with that of sinapic acid. The presence of sinapic acid derivatives in rapeseed has been reported in the literature [43]. Among them, more polar compounds than sinapic acid, including sinaopyl glucose and sinaopyl malate, and less polar compounds, such as disinapic acid and its derivatives, were found. In our study, two sinapic acid derivatives (compounds **2** and **3**) were also more polar, and two others (compounds **5** and **6**) were eluted after sinapic acid. However, further research on determining the structure of these compounds using more advanced analytical techniques and their full identification is necessary. Ferulic acid and its derivatives, quercetin-sinapoyl di-hexosepentose, and kaempferol 3-sinapoylsophorotrioside-7-glucoside were another phenolic compound identified previously in rapeseeds [43]. Our study did not confirm the presence of these compounds in the analyzed varieties.

The effect of different fertilization systems used in our study on the content of total and individual phenolic compounds and the antioxidant potential of the rapeseeds and their extracts varied depending on the plant variety and antioxidant assay; however, clear trends of change were not found. The results of scientific research also do not indicate a clear effect of fertilization on the content of phenolic compounds in rapeseed and other plants belonging to the Brassicaceae family and their antioxidant potential. Increased nitrogen fertilization of rapeseed, as claimed by Butkutė et al. [22] and Mert-Türk et al. [39], increased the content of phenolic compounds in seeds. De Pascale et al. [44] demonstrated an increased accumulation of phenolic acids, including gallic acid, in *Brassica rapa* plants under the influence of increased sulfur fertilization. Fertilization of red cabbage with 150 kg N/ha (nitrogen in the form of ammonium nitrate) was most affected in terms of the content of phenolic compounds and the antioxidant capacity [45]. Sady et al. [46] found the highest content of phenolics in *Brassica oleracea* var. *capitata* alba L. fertilized with RSM (solution ammonium nitrate and urea 1:1). In contrast, according to Li et al. [47], a significant decrease in phenolic compounds in mustard leaves was caused by increasing the level of nitrogen fertilization. Broccoli showed a decrease in flavonoid contents with nitrogen fertilization [48]. The maximum concentration of phenolic compounds was found in broccoli [49] and Chinese cabbage [50] in nonfertilized N plants.

In the ecological system used for *Brassica rapa* L. chinensis plant cultivation, the level of phenolic compounds was higher compared to the conventional system [51]. Johnson et al. [52] reported that a liquid organic nitrogen source should be applied rather than traditional mineral fertilization due to the increased accumulation of *p*-coumaric acid in pac choi. In the organic growing cauliflower *Brassica oleracea* L. subsp. Botrytis, compared to a conventional one, 24, 21, 13, 48, and 44% higher contents of ascorbic acid, polyphenols, carotenoids, and volatile substances were found, as well as a higher antioxidant capacity [53]. Conversa et al. [54] also showed a higher level of bioactive compounds in organic *Brassica rapa* L. The influence of different nitrogen fertilizations at doses of 0, 100, 200, and 300 mg/dm^3^, as well as the type of soil, did not affect the total phenolic content and FRAP results in the two varieties of kale, *Brassica oleracea* L. var. acephala [55]. However, red kale, in comparison with green-leafed kale, exhibited a positive effect of N fertilization. In addition, in other research, the production system did not show any effect on the total phenolic content and total flavonoid content in cauliflower (except for single genotypes) [56] or broccoli [57]. In experiments by Conversa et al. [54], the modifying effect of conventional and organic growing systems on the antioxidant properties of crude or processed *Brassica rapa* L. subsp. Sylvestris was not observed. According to Ibrahim and Jaafar [58], Zhang et al. [59], and Deng et al. [60], nitrogen deficiency or lower nitrogen content can trigger ethylene signaling and upregulate the transcription factor MYB12 to stimulate genes involved in the biosynthesis of phenylpropanoids and flavonoids, including chalcone synthase and L-phenylalanine ammonia-lyase. The results of a meta-analysis by Sun et al. [61] suggest a reduction in the internal C/N ratio by nitrogen application and a lowering of the biosynthesis of phenolic compounds, such as phenolic acids, flavonoids, including anthocyanins, and condensed tannins. Regulation by N at the branch point of 3-dehydroshikimate is proposed by Salminen and Maarit [28] to explain the variation in the abundance pattern of phenylpropanoids.

The principal component analysis (PCA) was employed in our study to further understand the relationship within the system. For the data relating to extracts, the importance of PC2 was relatively low (15.02%), and PC1 explained the majority of the variance (77.43%). This significant component discriminated the samples according to the variety rather than the type of fertilization, although in the case of the Nelson F1 variety, the division into seeds from different cultivations occurred along PC1. Considering the PCA results for the seed data, both PC1 (60.25%) and PC2 (36.41) significantly explained the total variance, and they clearly discriminated the Castilla variety from the remaining varieties. For the California and Nelson F1 seeds, samples were discriminated along PC1 and PC2, but no clear clusters were observed based on cultivation type, so we concluded that the effect of fertilization was individual for each variety.

The data from the PCA and the Pearson correlation analysis showed that the results of total phenolics, ABTS, and FRAP assays correlated with each other. The same relation between similar variables was observed for false flax extracts [38] and extracts from seeds of different *Vitis* species [62].

## 5. Conclusions

This study delved into the impact of varied fertilization techniques on the phenolic compound profile and antioxidant activity of different rapeseed cultivars. We observed significant differences in phenolic content, with the California variety showing the highest content under control conditions. Intensive fertilization reduced the content of phenolic compounds in the Nelson F1 seeds, while economic fertilization decreased the phenolic content in the California variety. Antioxidant assessments through ABTS and FRAP assays revealed cultivar-specific responses. The California variety exhibited higher antioxidant activity in intensively cultivated seeds, whereas the Nelson F1 variety showed stronger antiradical activity in spare-cultivated seeds. HPLC analysis identified key phenolic compounds, predominantly sinapine and sinapic acid. These findings highlight the complex relationship between fertilization practices, phenolic compound accumulation, and the antioxidant activity of rapeseed. Integrating varietal traits and cultivation practices is crucial for optimizing the nutritional benefits of rapeseed. Future research should explore the biochemical mechanisms influencing phenolic compound synthesis in response to specific fertilization regimens.

## Figures and Tables

**Table 1:** Schema of fertilization (kg/ha) during the field experiment.

Fertilization	Experimental Group		
	Control	Intensive	Spare
Phosphorus: Autumn	60	80	40
Potassium: Autumn	120	150	60
Nitrogen: Autumn	30	30	30
Nitrogen: Spring I	120	120	120
Nitrogen: Spring II	60	80	40
Sulphur: Spring	45	60	-

**Table 2:** Effect of fertilization on the content of total phenolics and antioxidant potential determined as Trolox equivalent antioxidant capacity (TEAC) and ferric-reducing antioxidant potential (FRAP) of rapeseed fresh matter (FM) and extracts.

Assay	Unit	Cultivar	Experimental Group		
			Control	Intensive	Spare
Total Phenolics	mg SAE/g extract	California	60.9 ± 2.6 ab	59.5 ± 0.7 a	57.1 ± 0.8 b
		Castilla	48.9 ± 1.9 ab	48.3 ± 0.4 a	49.4 ± 0.5 b
		Nelson F1	53.9 ± 0.4 a	49.8 ± 1.2 b	53.2 ± 0.3 a
	mg SAE/g seed FM	California	5.21 ± 0.22 a	5.16 ± 0.06 a	4.60 ± 0.06 b
		Castilla	4.49 ± 0.17 a	4.52 ± 0.12 a	4.47 ± 0.05 a
		Nelson F1	6.04 ± 0.04 a	5.18± 0.12 b	5.26 ± 0.03 b
TEAC	mmol TE/g extract	California	0.468 ± 0.039 a	0.417 ± 0.035 a	0.469 ± 0.035 a
		Castilla	0.366 ± 0.033 a	0.360 ± 0.033 a	0.365 ± 0.032 a
		Nelson F1	0.411 ± 0.015 a	0.418 ± 0.020 a	0.432 ± 0.015 a
	mmol TE/g seed FM	California	0.040 ± 0.003 a	0.036 ± 0.003 a	0.038 ± 0.003 a
		Castilla	0.034 ± 0.003 a	0.034 ± 0.003 a	0.033 ± 0.003 a
		Nelson F1	0.033 ± 0.003 b	0.043 ± 0.002 a	0.043 ± 0.002 a
FRAP	mmol Fe^2+^/g extract	California	1.61 ± 0.03 a	1.66 ± 0.05 a	1.43 ± 0.05 b
		Castilla	1.34 ± 0.0 a	1.30 ± 0.06 a	1.30 ± 0.08 a
		Nelson F1	1.35 ± 0.11 a	1.15 ± 0.05 b	1.31 ± 0.04 a
	mmol Fe^2+^/g seed FM	California	0.138 ± 0.003 a	0.144 ± 0.004 a	0.115 ± 0.004 b
		Castilla	0.123 ± 0.007 a	0.122 ± 0.006 a	0.122 ± 0.007 a
		Nelson F1	0.151 ± 0.012 a	0.120 ± 0.005 b	0.129 ± 0.004 b

Means with different letters in the same row are significantly different (*p* < 0.05). SAE—sinapic acid equivalent; TE—Trolox equivalent.

**Table 3:** Effect of fertilization on the content of individual phenolic compounds in rapeseed extracts and seeds.

Compound	Unit	Cultivar	Experimental Group		
			Control	Intensive	Spare
1 (Sinapine)	mg/g extract	California	80.1 ± 1.4 a	81.9 ± 5.1 a	76.2 ± 4.0 a
		Castilla	76.3 ± 3.4 a	73.0 ± 5.6 a	70.1 ± 1.4 a
		Nelson F1	65.8 ± 3.0 a	64.5 ± 3.3 a	70.8 ± 5.3 a
	mg/g seed FM	California	6.8 ± 0.1 a	7.0 ± 0.4 a	6.2 ± 0.3 a
		Castilla	7.0 ± 0.3 a	6.8 ± 0.5 a	6.3 ± 0.1 a
		Nelson F1	7.4 ± 0.3 a	6.7 ± 0.3 a	7.0 ± 0.5 a
2 (Sinapic acid derivative)	mg/g extract	California	8.4 ± 1.4 a	7.7 ± 0.6 a	7.6 ± 0.6 a
		Castilla	7.1 ± 0.9 a	7.1 ± 1.2 a	6.4 ± 0.2 a
		Nelson F1	4.0 ± 0.1 a	2.5 ± 0.2 b	3.1 ± 0.8 ab
	mg/g seed FM	California	0.72 ± 0.03 a	0.67 ± 0.05 a	0.61 ± 0.05 a
		Castilla	0.65 ± 0.08 a	0.66 ± 0.11 a	0.58 ± 0.02 a
		Nelson F1	0.45 ± 0.01 a	0.26 ± 0.02 b	0.31 ± 0.08 b
3 (Sinapic acid derivative)	mg/g extract	California	5.6 ± 0.3 a	5.1 ± 0.2 a	5.1 ± 0.3 a
		Castilla	5.9 ± 0.5 a	5.9 ± 1.0 a	5.4 ± 0.5 a
		Nelson F1	5.2 ± 0.1 a	4.9 ± 0.2 a	5.0 ± 0.6 a
	mg/g seed FM	California	0.48 ± 0.03 a	0.44 ± 0.02 a	0.41 ± 0.02 a
		Castilla	0.54 ± 0.06 a	0.49 ± 0.06 a	0.50 ± 0.01 a
		Nelson F1	0.58 ± 0.01 a	0.51 ± 0.02 b	0.50 ± 0.04 b
4 (Sinapic acid)	mg/g extract	California	6.3 ± 0.4 a	6.2 ± 0.2 a	6.1 ± 0.3 a
		Castilla	5.9 ± 0.7 a	5.2 ± 0.6 a	5.5 ± 0.1 a
		Nelson F1	3.8 ± 0.2 a	3.9 ± 0.5 a	4.4 ± 0.8 a
	mg/g seed FM	California	0.54 ± 0.04 a	0.54 ± 0.03 a	0.49 ± 0.02 a
		Castilla	0.54 ± 0.06 a	0.49 ± 0.06 a	0.50 ± 0.01 a
		Nelson F1	0.43 ± 0.02 a	0.41 ± 0.05 a	0.44 ± 0.08 a
5 (Sinapic acid derivative)	mg/g extract	California	-	-	-
		Castilla	-	-	-
		Nelson F1	18.3 ± 0.5 a	16.9 ± 1.0 a	17.1 ± 0.9 a
	mg/g seed FM	California	-	-	-
		Castilla	-	-	-
		Nelson F1	2.05 ± 0.06 a	1.76 ± 0.01 b	1.69 ± 0.09 b
6 (Sinapic acid derivative)	mg/g extract	California	8.5 ± 0.5 a	8.3 ± 0.2 a	7.6 ± 0.5 a
		Castilla	6.0 ± 0.7 a	5.7 ± 0.2 a	6.4 ± 1.5 a
		Nelson F1	3.8 ± 0.4 a	3.2 ± 0.4 a	3.5 ± 0.8 a
	mg/g seed FM	California	0.73 ± 0.04 a	0.72 ± 0.02 a	0.61 ± 0.04 a
		Castilla	0.55 ± 0.05 a	0.53 ± 0.05 a	0.58 ± 0.05 a
		Nelson F1	0.43 ± 0.04 a	0.33 ± 0.04 b	0.35 ± 0.08 b

Means with different letters in the same row are significantly different (*p* < 0.05). FM—fresh matter.

**Table 4:** Pearson correlations between total phenolic content, Trolox equivalent antioxidant capacity (TEAC), ferric-reducing antioxidant potential (FRAP), and DPPH radical scavenging activity of rapeseed of different varieties cultivated in different conditions.

Variables	Regression Equation	Correlation Coefficient
Total phenolics vs. TEAC	y = 0.0071x + 0.0327	0.814 *
Total phenolics vs. FRAP	y = 0.0293x + 0.5733	0.871 *
Total phenolics vs. DPPH	y = −0.0027x + 0.4418	−0.271
TEAC vs. FRAP	y = 1.740x + 0.667	0.672 *
TEAC vs. DPPH	y = −0.2182x + 0.3864	−0.190
FRAP vs. DPPH	y = −0.076x + 0.4016	−0.255

* significant at *p* < 0.05.

## Data Availability

Data is contained within the article.
